# Nanoformulations for Ocular Drug Delivery in Glaucoma: From Metal-Based Nanoparticles to Advanced Nanocarriers

**DOI:** 10.3390/pharmaceutics18091181

**Published:** 2026-09-18

**Authors:** Ionut Iulian Lungu, Liviu Stafie, Alina Stefanache, David-Constantin Stefan, Andreea Lungu, Georgiana-Elena Sarbu, Liliana Savin, Andreea Guraliuc, Francesca Cristiana Dohotariu, Nicoleta Anton

**Affiliations:** 1 Grigore T. Popa University of Medicine and Pharmacy Iasi, 700115 Iasi, Romania; ionut-iulian.lungu@umfiasi.ro (I.I.L.); liviu.stafie@umfiasi.ro (L.S.); alina.stefanache@umfiasi.ro (A.S.);; 2 Institute of Gastroenterology and Hepatology, “St. Spiridon” Emergency County Hospital, 700111 Iasi, Romania; 3 Department Orthopedics and Traumatology, Clinical Rehabilitation Hospital, 700661 Iasi, Romania; 4 Ophthalmology Clinic, Sf. Spiridon Emergency Clinical Hospital, 700111 Iasi, Romania

**Keywords:** glaucoma, nanoformulation, metal nanoparticles, ocular drug delivery, nanomedicine, intraocular pressure, neuroprotection

## Abstract

Glaucoma is a chronic and progressive optic neuropathy in which sustained intraocular pressure (IOP) reduction remains the main therapeutic objective, while increasing attention is being directed toward neuroprotective strategies and improved ocular drug delivery. Conventional topical therapies are frequently limited by short precorneal residence time, poor ocular bioavailability, and the need for frequent administration. This narrative review examines nanoformulation-based strategies investigated for ocular drug delivery in glaucoma, ranging from metal-based nanoparticles, including gold, silver, zinc-, copper-, and iron-based systems, to advanced nanocarriers such as liposomes, polymeric nanoparticles, solid lipid nanoparticles, nanostructured lipid carriers, nanoemulsions, nanosuspensions, dendrimers, cyclodextrin-based systems, and hydrogels. Particular attention is given to the relationship between nanocarrier composition, physicochemical characteristics, drug-loading and release properties, ocular retention and penetration, therapeutic effects, and the level of experimental evidence. The review further compares metal-based nanoparticles with non-metal-based nanocarriers, highlighting their respective advantages and limitations in terms of drug delivery, sustained ocular exposure, therapeutic potential, biocompatibility, toxicity, and translational relevance. Although several nanoformulation platforms have demonstrated promising results in preclinical studies, the available evidence remains heterogeneous and predominantly preclinical. Further research is needed to establish long-term ocular safety, optimize formulation characteristics, standardize evaluation methods, and determine the clinical relevance of these emerging delivery systems.

## 1. Introduction

Glaucoma is a chronic and progressive optic neuropathy characterized by retinal ganglion cell degeneration, structural damage to the optic nerve head, and corresponding visual field loss. Although elevated intraocular pressure (IOP) remains the major modifiable risk factor, increasing evidence suggests that glaucoma is a multifactorial neurodegenerative disease involving oxidative stress, mitochondrial dysfunction, vascular dysregulation, and neuroinflammatory pathways. These mechanisms contribute to retinal ganglion cell apoptosis and may explain disease progression even in patients with adequately controlled IOP [1,2,3,4].

Glaucoma is one of the leading causes of irreversible blindness worldwide and represents a major public health concern. The global prevalence of the disease is expected to increase substantially in the coming decades due to population aging and increasing life expectancy. Beyond vision loss, glaucoma is associated with reduced quality of life, impaired daily functioning, and significant socioeconomic burden [5,6,7].

One of the major challenges in glaucoma management is the asymptomatic nature of the disease during its early stages. Structural and functional damage may occur years before patients become aware of visual impairment, resulting in delayed diagnosis and treatment initiation. Furthermore, disease progression may continue despite successful IOP reduction, highlighting the need for therapeutic approaches that target neurodegeneration, oxidative stress, and neuroinflammation in addition to pressure control [3,8,9].

Despite significant advances in glaucoma diagnosis and management, current therapeutic approaches remain largely focused on lowering intraocular pressure, which is currently the only clinically proven modifiable risk factor. However, growing evidence indicates that glaucoma progression is driven by multiple pathogenic mechanisms, including retinal ganglion cell apoptosis, neuroinflammation, oxidative stress, mitochondrial dysfunction, and vascular impairment. Consequently, there is increasing interest in identifying novel therapeutic targets capable of providing neuroprotection and preserving visual function beyond conventional pressure-lowering strategies. A deeper understanding of the molecular and cellular pathways involved in glaucoma pathogenesis is therefore essential for the development of more effective and personalized treatment approaches [1,2,3,4].

Topical eye drops continue to represent the most commonly used pharmaceutical dosage form for glaucoma treatment. However, conventional aqueous eye drops suffer from severe precorneal loss, where over 95% to 99% of the instilled dose is eliminated within 90–120 s following administration. This rapid loss is driven by reflexive blinking, nasolacrimal drainage, and continuous physiological tear turnover (0.5–2.2 μL/min), leaving only an extremely brief window for transcorneal absorption [10,11]. Consequently, nanoformulation-based approaches have attracted considerable interest as an engineered solution to overcome these pharmacokinetic bottlenecks [12,13,14,15]. By engineering delivery platforms at the nanoscale (50–200 nm), these systems prolong precorneal residence time through bioadhesive interactions with corneal and conjunctival mucins, provide controlled release kinetics that minimize peak–trough IOP fluctuations, protect labile hydrophobic compounds from enzymatic or chemical degradation, and facilitate transcorneal permeation via intracellular endocytic pathways [10,12,16]. A wide range of platforms has been investigated, including liposomes, polymeric and lipid-based nanoparticles, nanoemulsions, dendrimers, and hydrogels [14,15]. Metal-based nanomaterials represent a distinct category because they may provide additional functionalities, including imaging capability, magnetically assisted delivery, surface functionalization, and modulation of oxidative or neuroprotective pathways [13,14,17,18,19]. However, their potential benefits must be considered alongside unresolved issues related to ocular safety, tissue accumulation, formulation reproducibility, manufacturing complexity, and the still limited amount of clinical evidence [12,13,14,18,20].

A critical prerequisite for ocular particulate systems is the strict optimization of particle size, which governs ocular tolerability and cellular transport pathways [10,12]. In ophthalmic administration, the physical comfort threshold is defined by particle dimensions below 10–20 μm; particles exceeding this diameter induce mechanical irritation, foreign-body sensation, and reflexive tearing that accelerates formulation wash-out [10,21]. Conversely, reducing carrier dimensions to the sub-micron and nanoscale regimen (50–200 nm) avoids sensory stimulation and enables transcorneal permeation through receptor-mediated or adsorptive endocytosis, facilitating intracellular drug partitioning across the intact epithelium [10,12,16]. Within this framework, metallic nanoparticles represent a fundamentally distinct class of delivery systems beyond conventional organic polymers or lipids [13,14,22]. While standard nanocarriers serve primarily as passive transport reservoirs, metallic platforms offer multifunctional therapeutic and theranostic properties [13,14,19,22]. Specifically, their unique localized surface plasmon resonance (LSPR) and tunable surface chemistries permit high-density bioconjugation [17,22]. Furthermore, metallic nanomaterials can exert intrinsic biological activities, such as superoxide dismutase- and catalase-mimetic reactive oxygen species (ROS) scavenging (notably gold and cerium systems) to mitigate trabecular meshwork and retinal oxidative injury [17,23,24]. Additionally, superparamagnetic iron oxide nanoparticles provide thermal-stimuli responsiveness—inducing localized heat-shock protein 70 (HSP70) neuroprotective pathways via controlled magnetic hyperthermia—alongside magnetic field guidance and high-contrast imaging of trabecular outflow architecture and the optic nerve head [13,14,19].

This narrative review critically examines nanoformulation-based strategies investigated for ocular drug delivery in glaucoma, ranging from metal-based nanoparticles to advanced lipid, polymeric, dendritic, and hydrogel-based systems. Particular emphasis is placed on the relationship between nanocarrier composition, drug-delivery performance, therapeutic rationale, and level of experimental evidence. Representative preclinical findings are considered alongside formulation-specific limitations, ocular safety concerns, manufacturing challenges, and barriers to clinical translation. By comparing the principal nanoformulation classes within a common therapeutic framework, this review aims to identify both the most promising opportunities and the major evidence gaps that currently limit their incorporation into routine glaucoma management. In this context, the present review considers these nanoformulation strategies within a common framework of ocular drug delivery in glaucoma, while giving particular attention to metal-based nanoparticles and their potential advantages and limitations compared with non-metal-based nanocarriers. The comparison considers relevant formulation and therapeutic characteristics, including drug loading, controlled release, ocular retention and penetration, biocompatibility, toxicity, and the level of experimental evidence. This approach is intended to highlight both the specific potential of metal-based nanoplatforms and the advantages offered by alternative nanocarrier systems.

## 2. Glaucoma Pathophysiology and Therapeutic Targets

### 2.1. Pathophysiological Mechanisms

Glaucoma is a multifactorial neurodegenerative disorder characterized by progressive retinal ganglion cell (RGC) loss and optic nerve damage, ultimately leading to irreversible visual impairment. Although elevated intraocular pressure (IOP) remains the primary modifiable risk factor, the pathogenesis of glaucoma involves a complex interplay of mechanical, vascular, metabolic, and inflammatory mechanisms. Increased resistance to aqueous humor outflow through the trabecular meshwork results in elevated IOP, which exerts mechanical stress on the optic nerve head, particularly at the level of the lamina cribrosa. This mechanical deformation disrupts axonal transport, compromises neuronal signaling, and initiates a cascade of events that promote retinal ganglion cell degeneration [1,2,3].

Retinal ganglion cell death is considered a central event in glaucoma progression. Experimental and clinical studies have demonstrated that prolonged mechanical stress and impaired axonal transport lead to neurotrophin deprivation, mitochondrial dysfunction, and activation of apoptotic pathways. In addition, excessive glutamate release may induce excitotoxicity through overstimulation of N-methyl-D-aspartate (NMDA) receptors, resulting in calcium overload and neuronal injury. These mechanisms contribute to progressive retinal ganglion cell loss even in cases where intraocular pressure is adequately controlled [3,8,9].

**Figure 1:**
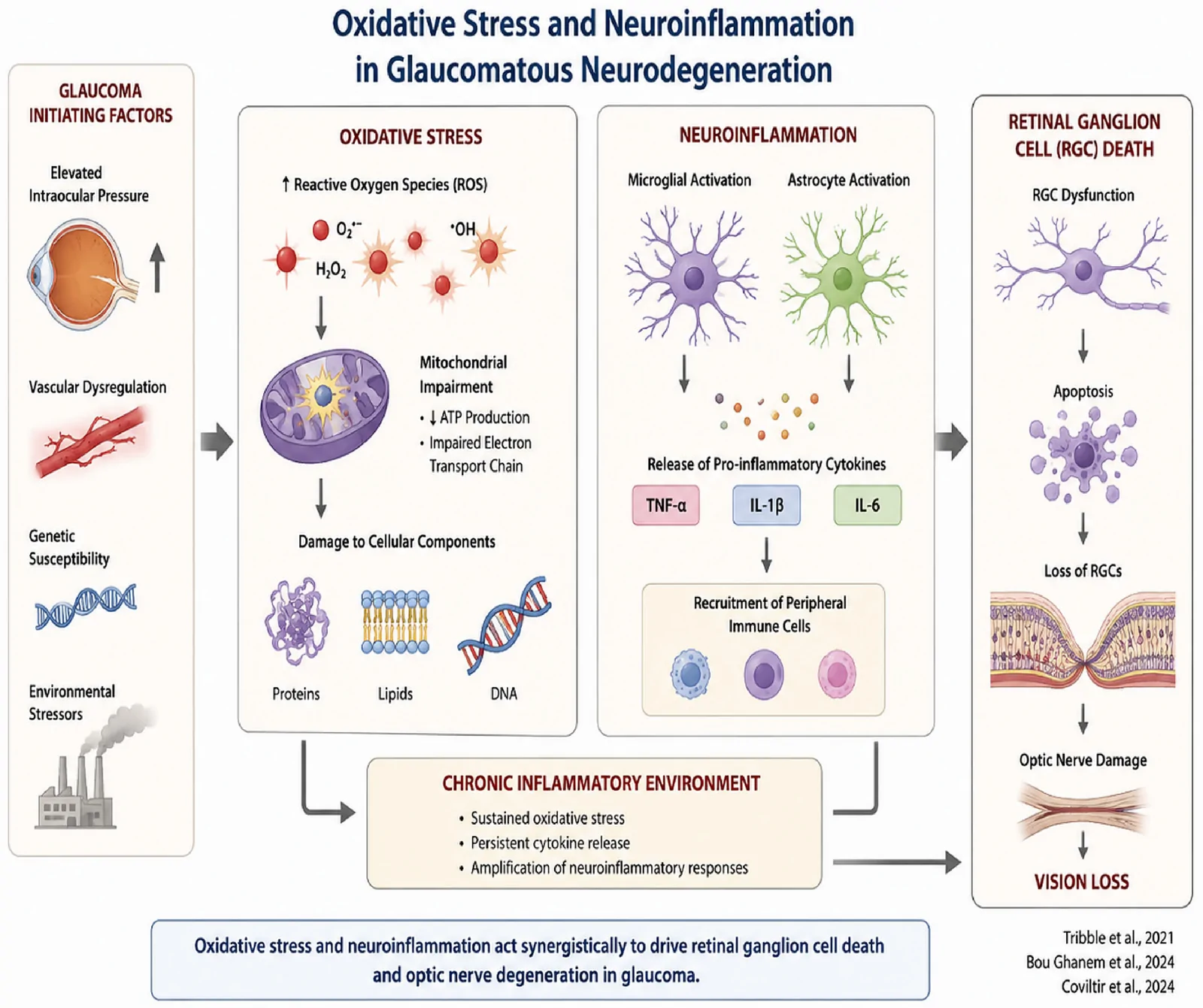
Oxidative stress and neuroinflammatory pathways contributing to glaucomatous neurodegeneration. References shown in the figure are cited here as [3,4,25]. Arrows indicate the direction of the described pathophysiological processes; the color coding distinguishes functional components.

Oxidative stress and neuroinflammation have emerged as key contributors to glaucomatous neurodegeneration (Figure 1). Elevated levels of reactive oxygen species (ROS) can damage cellular proteins, lipids, and DNA, while mitochondrial impairment further exacerbates energy depletion and neuronal vulnerability. Simultaneously, activation of microglia and astrocytes promotes the release of pro-inflammatory cytokines, including tumor necrosis factor-alpha (TNF-α), interleukin-1β (IL-1β), and interleukin-6 (IL-6), creating a chronic inflammatory environment that accelerates retinal ganglion cell death [3,4,25].

Vascular dysregulation also plays a significant role in glaucoma pathophysiology, particularly in patients with normal-tension glaucoma. Reduced ocular perfusion, endothelial dysfunction, and impaired autoregulation of blood flow may lead to recurrent ischemic episodes and insufficient nutrient supply to the optic nerve head. The growing recognition of these non-pressure-dependent mechanisms has transformed the understanding of glaucoma from a purely pressure-related disease into a complex neurodegenerative condition, thereby opening new opportunities for the development of targeted therapeutic interventions [2,4,26].

### 2.2. Therapeutic Targets

The primary goal of glaucoma treatment remains the reduction in intraocular pressure (IOP), which is currently the only proven modifiable risk factor associated with disease progression. Clinical trials have consistently demonstrated that lowering IOP significantly reduces the risk of visual field deterioration and slows optic nerve damage. Consequently, most available pharmacological therapies, including prostaglandin analogs, β-blockers, carbonic anhydrase inhibitors, and α2-adrenergic agonists, are designed to decrease aqueous humor production or facilitate its drainage. Despite their effectiveness, disease progression may still occur in some patients, highlighting the need for complementary therapeutic approaches [1,2,27].

Enhancing aqueous humor outflow through the conventional trabecular meshwork pathway and the uveoscleral route has become a major therapeutic focus [1,27]. In recent years, the clinical development of Rho kinase (ROCK) inhibitors (such as netarsudil, ripasudil, and fasudil) has provided novel pharmacological mechanisms to lower intraocular pressure by relaxing trabecular meshwork cells, modifying extracellular matrix deposition, and reducing outflow resistance across the juxtacanalicular tissue [28,29,30]. Despite this clear mechanistic advantage, conventional topical administration of ROCK inhibitors and first-line prostaglandin analogs (e.g., latanoprost, bimatoprost) faces major bioavailability constraints [10,11]. Rapid precorneal clearance and non-specific conjunctival absorption not only reduce drug partitioning into the anterior chamber but also provoke prominent off-target adverse effects, most notably severe conjunctival hyperemia and ocular surface irritation [27,28]. In this setting, particulate nanoformulations establish a direct functional link between target pharmacology and local bioavailability [12,16]. By encapsulating outflow-modulating agents within nanocarriers engineered for controlled release and sustained tissue interaction, nanoformulations maximize active drug delivery specifically to the trabecular meshwork and ciliary body while minimizing superficial conjunctival exposure and eliminating sharp peak-trough concentration swings [31,32].

Given that retinal ganglion cell degeneration represents the final common pathway leading to vision loss, neuroprotection has emerged as a promising therapeutic strategy. Neuroprotective approaches aim to preserve neuronal viability independently of IOP reduction by targeting mechanisms such as excitotoxicity, mitochondrial dysfunction, impaired neurotrophic support, and apoptosis. Experimental studies have investigated neurotrophic factors, NMDA receptor antagonists, mitochondrial protectants, and agents capable of enhancing cellular resilience under stress conditions. Although several candidates have shown encouraging preclinical results, their clinical translation remains an active area of research [3,8,9].

Increasing evidence also supports the therapeutic potential of targeting neuroinflammation and oxidative stress in glaucoma. Chronic activation of microglia and astrocytes contributes to the production of pro-inflammatory cytokines and reactive oxygen species, which further promote retinal ganglion cell injury. Consequently, anti-inflammatory agents, antioxidants, and therapies aimed at preserving mitochondrial function have gained considerable attention. Compounds such as nicotinamide, coenzyme Q10, and other mitochondrial-targeted interventions have demonstrated neuroprotective effects in experimental models and are currently being explored as adjunctive treatments. These emerging strategies reflect a broader shift toward addressing the multifactorial nature of glaucoma and developing treatments capable of slowing neurodegeneration beyond conventional pressure-lowering therapy [3,4,25,33].

## 3. Limitations of Conventional Ocular Drug Delivery

### 3.1. Ocular Barriers

The eye possesses multiple protective static and dynamic barriers that preserve ocular integrity but severely restrict drug penetration [10,11]. The corneal epithelium represents the primary rate-limiting barrier to topical drug absorption [10]. Its tight junctions (zonula occludens) exhibit an average functional pore radius of approximately 2.0 nm, strictly limiting the paracellular diffusion of hydrophilic molecules exceeding 500 Da [10,21]. Transcellular transport across the epithelium requires an optimal lipophilic balance, typically within a log P range of 1 to 3 [10]. Conversely, the transscleral route offers a wider delivery window via a fibrous, aqueous proteoglycan matrix featuring functional pores of 20–40 nm, enabling the permeation of larger macromolecules [10,21]. Nevertheless, because scleral proteoglycans carry a dense negative charge, cationic carriers undergo electrostatic entrapment, whereas neutral, PEGylated, or zwitterionic nanocarriers exhibit up to 10-fold greater transscleral flux [10,12,34]. To navigate these barriers efficiently, targeted surface-engineering strategies are essential [12,16]: (1) dense PEGylation (2–5 kDa)—creates a neutral, hydrophilic coating that prevents entrapment in precorneal mucins and accelerates diffusion across mucosal layers [12,32]; (2) cell-penetrating peptides (e.g poly-L-arginine)—facilitate direct translocation across epithelial lipid bilayers independently of classical endocytic pathways [12,14]; (3) CD44-targeting ligands (e.g., hyaluronic acid)—exploit receptor overexpression on reactive trabecular meshwork cells and injured retinal ganglion cells in glaucomatous eyes, enhancing site-specific uptake while mitigating off-target toxicity [16,35,36].

### 3.2. Limitations of Topical Therapy

Topical eye drops remain the most commonly prescribed treatment for glaucoma due to their non-invasive administration and favorable safety profile. Nevertheless, their clinical effectiveness is limited by extremely low ocular bioavailability. It is estimated that less than 5% of an administered topical dose successfully penetrates the cornea and reaches intraocular tissues, while the remaining fraction is lost through tear drainage, conjunctival absorption, or systemic circulation. This inefficiency often necessitates higher drug concentrations and repeated administration to achieve therapeutic efficacy [11,37].

Poor patient adherence represents one of the most significant barriers to successful glaucoma management. Numerous studies have demonstrated that a substantial percentage of patients fail to administer medications as prescribed due to forgetfulness, difficulties with eye drop instillation, complex treatment schedules, or treatment-related discomfort. Reduced adherence is strongly associated with inadequate intraocular pressure control and accelerated disease progression, ultimately increasing the risk of irreversible vision loss [27,37,38].

In addition to local limitations, topical administration may also result in systemic exposure through drainage into the nasolacrimal duct and subsequent absorption through the nasal mucosa. This pathway bypasses first-pass hepatic metabolism and may contribute to systemic adverse effects, particularly in patients receiving β-blockers such as timolol. Reported systemic complications include bradycardia, hypotension, bronchospasm, and other cardiovascular or respiratory effects, highlighting the need for safer and more targeted ocular drug delivery systems [11,27,39].

Overall, the numerous anatomical, physiological, and patient-related limitations associated with conventional ocular drug delivery have driven the development of advanced dosage forms designed to improve bioavailability, prolong drug residence time, enhance patient compliance, and provide sustained therapeutic effects. These emerging approaches include nanoparticles, lipid-based carriers, hydrogels, drug-eluting contact lenses, microneedles, and biodegradable implants, which are increasingly being investigated as promising alternatives for glaucoma therapy [37]. (Table 1).

## 4. Conventional Ocular Drug Delivery Systems

Conventional ocular dosage forms are traditionally categorized by their physical state into aqueous solutions, aqueous suspensions, coarse emulsions, and semisolid ointments [10,21,40]. Aqueous solutions (standard eye drops) offer immediate dissolution and simple instillation, but undergo complete precorneal clearance within 90–120 s, yielding less than 5% bioavailability [10,41]. To accommodate poorly soluble lipophilic compounds, aqueous macro-suspensions and coarse emulsions are utilized [21,42]. However, suspensions remain thermodynamically unstable and prone to sedimentation and caking, which causes dosing heterogeneity with repeated use, while coarse suspended microparticles induce ocular discomfort and reflex tearing [21,40]. Coarse emulsions suffer from droplet coalescence, phase separation, and surfactant-induced irritation [43]. Semisolid ointments markedly prolong residence time, but cause transient visual blurring due to refractive disruption of the tear film, restricting their use to bedtime [44]. Nanoscale pharmaceutical engineering resolves these specific physicochemical deficits: nanosuspensions accelerate dissolution velocity without foreign-body sensation, nanoemulsions provide thermodynamic stability with reduced surfactant loads, and in situ gelling systems deliver extended contact time without refractive interference [12,37,45].

## 5. Metal-Based Nanoparticles in Glaucoma Therapy

Metal-based nanoparticles have attracted growing interest as ocular drug delivery platforms owing to their distinct surface and structural characteristics, such as high surface-area-to-volume ratio, tunable particle size, and the possibility of surface functionalization, which make them versatile carriers for ocular therapeutics. An optimal particulate drug delivery platform for ophthalmic therapy must satisfy rigorous physicochemical and biological criteria: (1) a strictly monodisperse hydrodynamic diameter between 50 and 200 nm to facilitate transcorneal endocytosis while remaining well below the 0.5–2.0 μm pore clearance threshold of the human trabecular meshwork [10,12]; (2) a moderately positive zeta potential (+10 to +30 mV) or a dense neutral polyethylene glycol (PEG) shell to enable mucopenetration without causing cytotoxic epithelial membrane disruption [12,16]; (3) sustained release profiles that maintain stable intraocular drug levels over extended intervals [14,31]; (4) complete biocompatibility with non-toxic degradation pathways [14,46]. The inorganic and metal-based nanoparticles possess advantageous features as we mentioned above, their clinical viability must be evaluated against these benchmark parameters, particularly regarding non-biodegradability and chronic intraocular retention [14,17,22,46].

Gold, silver, zinc, copper, and iron oxide nanoparticles have each been investigated for potential applications in glaucoma management, ranging from drug delivery and imaging to intrinsic antioxidant and neuroprotective activity [22].

### 5.1. Gold Nanoparticles

In ocular drug delivery, gold nanoparticle (AuNP)-containing contact lenses have been investigated as an alternative to conventional topical administration, particularly for prolonging drug release. Li et al. reported that incorporation of gold nanoparticles into the contact-lens matrix enhanced bimatoprost uptake and enabled sustained in vitro drug release for up to 72 h, while improving oxygen permeability and optical transmittance and reducing protein adherence [47]. This result indicates that the nanoparticle-containing lens can extend drug exposure beyond that achieved with conventional topical administration.

The influence of AuNP morphology on ocular drug delivery has also been investigated in glaucoma. In a rabbit glaucoma model, PLGA-coated gold nanospheres and nanorods loaded with a carbonic anhydrase inhibitor produced greater IOP reduction than the corresponding marketed eye drops. Notably, spherical AuNPs showed superior efficacy compared with nanorods, which was attributed to enhanced retention within the corneal stroma. Histological examination revealed no apparent corneal or retinal abnormalities following treatment with the spherical formulation, suggesting favorable short-term ocular tolerability [48]. It is important to critically note, however, that the pressure-lowering effect in this model was driven by the released carbonic anhydrase inhibitor payload rather than by the gold core itself; the AuNP functioned primarily as an electron-dense, rigid structural template that influenced PLGA shell deposition and geometric curvature, without contributing intrinsic hypotensive activity. Incorporating a non-biodegradable metallic core in this way introduces additional regulatory, toxicological, and long-term clearance considerations that are not offset by pharmacological synergy, and the supporting evidence remains limited to small preclinical cohorts (*n* = 4–6 rabbits) evaluated over acute time frames.

Gold nanoparticles have also demonstrated significant anti-inflammatory and antioxidant properties that contribute to the reduction in oxidative stress and inflammation, which are key factors involved in retinal degeneration and other ocular pathologies. Additionally, AuNPs have shown antimicrobial activity and can be incorporated into ophthalmic formulations to reduce the risk of postoperative infections [17]. Primary experimental evidence supports anti-inflammatory and antioxidant effects of gold nanoparticles in ocular models. In endotoxin-induced uveitis in rats, topical AuNPs reduced TNF-α, myeloperoxidase activity, oxidative tissue damage, and TLR4/NF-κB signaling [49]. In a separate study, hyaluronic-acid-coated AuNPs showed protective and antiangiogenic effects in retinal experimental models, including inhibition of AGE-mediated retinal pigment epithelial cell death and neovascularization [50].

Beyond surface-coated nanospheres, primary experimental studies provide direct evidence supporting the utility of AuNPs both as intracameral targeting vehicles and as diffusion-modulating reservoirs in therapeutic contact lenses. Specifically, Sonntag et al. [51] evaluated the intracameral distribution of AuNPs within the anterior chamber, demonstrating that sub-100 nm gold nanoparticles are transported via convective aqueous humor flow directly toward the iridocorneal angle, where they are efficiently internalized by trabecular meshwork endothelial cells without inducing acute cytotoxic detachment. Addressing surface engineering and colloidal mobility, Apaolaza et al. [52] demonstrated that surface coating of AuNPs with hyaluronic acid (HA) dramatically modifies their zeta potential, prevents non-specific protein corona adsorption, and facilitates ocular distribution, leveraging CD44 receptor-mediated interactions for targeted intraocular drug delivery while reducing cellular toxicity. In parallel, Maulvi et al. [50] formulated timolol-loaded hydrogel contact lenses incorporating embedded AuNPs; the inclusion of gold nanoparticles significantly augmented timolol uptake and extended drug release kinetics beyond 72 h in vitro without compromising lens optical transparency. In vivo evaluation in rabbits revealed that AuNP-laden lenses maintained sustained intraocular pressure reduction significantly longer than conventional timolol eye drops, providing direct experimental validation for gold-assisted sustained release systems.

**Figure 2:**
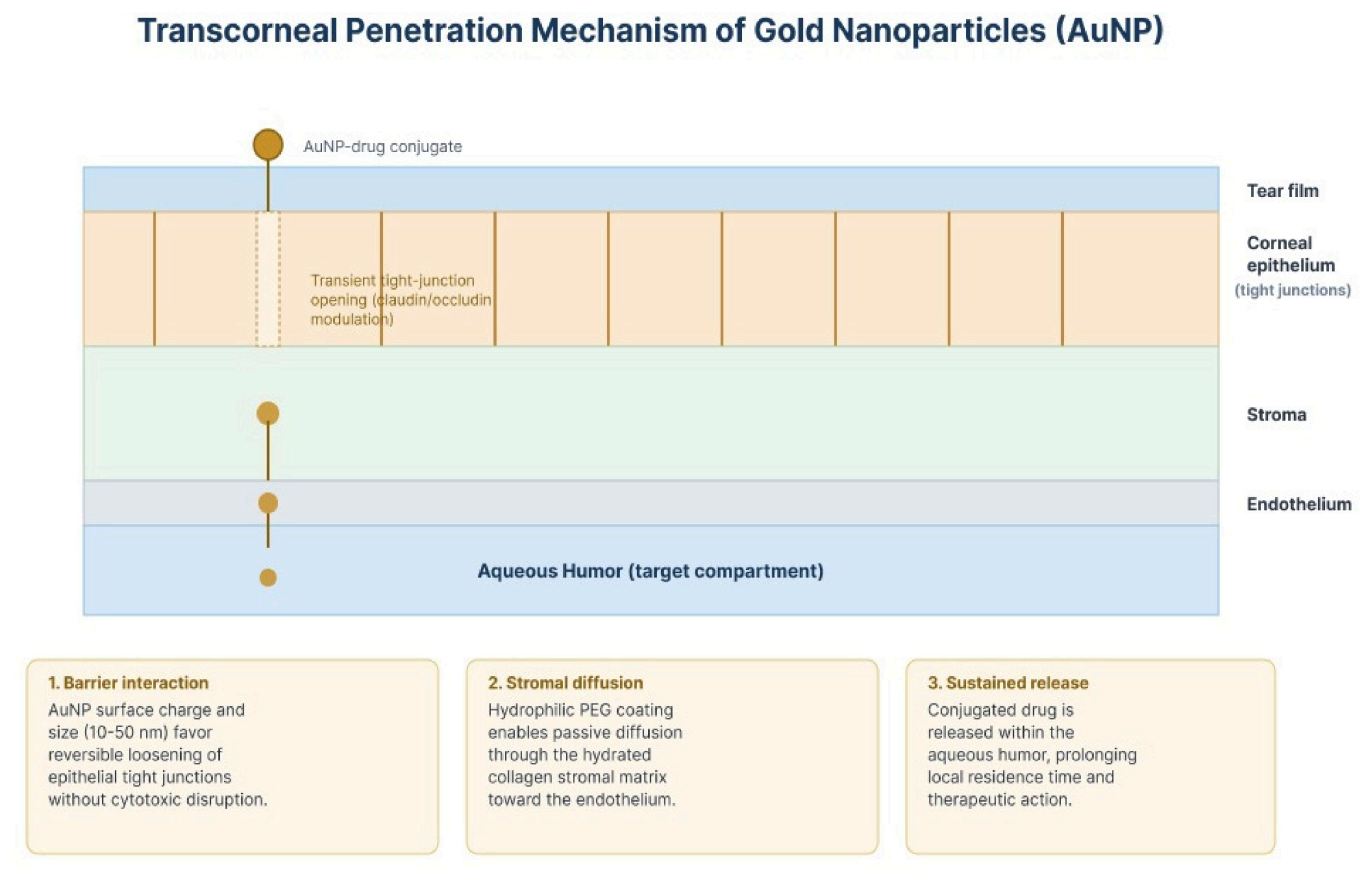
Transcorneal penetration pathway of gold nanoparticle (AuNP)-based drug conjugates. Color coding distinguishes the anatomical compartments, and arrows indicate the direction of nanoparticle movement across the ocular barriers.

To achieve stable covalent loading of antiglaucoma compounds onto metallic cores (as schematized in Figure 2), three principal bioconjugation chemistries are employed: (1) dative gold-thiol (Au-S) coordination bonds, utilizing thiolated ligands or cysteine residues to form self-assembled monolayers directly on the gold surface [22,48]; (2) EDC/NHS [1-ethyl-3-(3-dimethylaminopropyl)carbodiimide/N-hydroxysuccinimide] zero-length crosslinking, which covalently couples carboxyl-bearing coatings (e.g., thiolated PEG-COOH) to primary amine groups on peptide or drug payloads [16,17]; and (3) stimuli-cleavable linkers, such as pH-sensitive hydrazone or ester bridges that remain stable in the precorneal tear film but undergo hydrolytic cleavage within acidic endosomes or upon exposure to intraocular esterases, releasing the pharmacologically active payload [12,14,53].

### 5.2. Silver Nanoparticles

The biomedical interest in silver nanoparticles (AgNPs) is largely related to their antimicrobial and anti-inflammatory properties, together with their distinctive nanoscale physicochemical characteristics. Their high surface-area-to-volume ratio and optical behavior have supported investigation in drug delivery, diagnostics, biomaterials, and other biomedical applications.

Hasan et al. described protein-functionalized AgNPs as a modified approach to conventional silver nanomaterials, emphasizing the contribution of the protein component to biocompatibility, biodegradability, and surface functionalization. The review further discussed the possibility of using protein matrices to influence drug loading, release, and biodistribution, while potentially reducing some toxicity associated with unmodified AgNPs [54].

However, current glaucoma-focused reviews provide little direct preclinical evidence supporting AgNP-mediated IOP reduction or retinal ganglion cell neuroprotection. Both AgNPs and gelatin-functionalized AgNPs demonstrated antiangiogenic activity in the chorioallantoic membrane assay and in an experimental rabbit model of corneal neovascularization [15].

For AgNPs, an important concern is ocular toxicity. AgNP-induced cellular injury appears to depend strongly on particle size, concentration, surface chemistry, and exposure duration. In vitro studies have reported dose-dependent mitochondrial dysfunction and apoptosis in retinal pigment epithelial cells, while broader ocular nanotoxicology literature emphasizes the need for careful assessment of tissue accumulation and long-term retinal safety. These considerations are particularly important in glaucoma, where treatment is chronic and repeated exposure would be expected [46].

### 5.3. Copper-Based Nanoparticles

While copper nanoparticles (CuNPs) and copper oxide nanostructures possess antimicrobial and catalytic attributes in vitro [18], their ophthalmic application in glaucoma is severely limited by pronounced nanotoxicological liabilities [18,20]. Free cupric (Cu^2+^) and cuprous (Cu^+^) ions rapidly undergo Fenton-likeHaber–Weiss catalytic cycling, generating uncontrolled amounts of hydroxyl radicals (•OH) and superoxide anions [18,20]. Experimental nanotoxicological investigations demonstrate that copper oxide nanoparticles trigger severe mitochondrial membrane depolarization, deplete intracellular glutathione, upregulate caspase-3/9 apoptotic cascades, and provoke massive microglial activation [20,46]. Given that oxidative stress, mitochondrial failure, and neuroinflammation are key drivers of retinal ganglion cell loss and trabecular meshwork degeneration in glaucoma [3,4,25], unrestrained copper ion release would exacerbate intraocular tissue destruction rather than provide therapeutic benefit [20]. Thus, CuNPs are clinically unviable for chronic glaucoma pharmacotherapy unless enclosed within impermeable, non-leaching, biocompatible encapsulation matrices [14,18,20].

### 5.4. Iron Oxide Nanoparticles

An early example of nanoparticle-based neuroprotection was reported by Jeun et al., who investigated Mn_0.5_Zn_0.5_Fe_2_O_4_ nanoparticles as magnetic hyperthermia agents rather than conventional drug carriers. The approach was intended to induce localized heat-shock protein expression and activate endogenous cellular defense mechanisms, providing a proof-of-concept for magnetic nanoparticle-mediated neuroprotection in glaucoma [19].

A particularly valuable aspect of this review is its discussion of iron oxide nanoparticles as multifunctional therapeutic platforms rather than simple drug carriers. Magnetic nanoparticles can induce heat shock proteins through controlled magnetic hyperthermia, thereby activating the eye’s own neuroprotective mechanisms instead of relying solely on pharmacological agents. This concept represents a shift in glaucoma research, where neuroprotection is viewed as an essential complement to intraocular pressure reduction. Future glaucoma therapies will likely integrate sustained drug delivery with neuroprotective strategies, positioning nanotechnology, and particularly magnetic nanoparticles, as a promising component of next-generation ophthalmic treatments [13].

Zhai et al. provide a balanced overview of the current landscape of nanomedicine for glaucoma while also identifying the challenges that must be overcome before these technologies can reach clinical practice. Rather than focusing only on individual nanoparticle systems, the authors compare different nanoplatforms according to their therapeutic goals, including sustained drug delivery, intraocular pressure control, neuroprotection, gene delivery, and regenerative approaches.

Within this context, iron oxide nanoparticles are presented as particularly attractive because of their magnetic properties, which enable targeted delivery, external guidance, and potential integration with diagnostic imaging. However, the authors also emphasize that evidence supporting their use in glaucoma remains largely preclinical. By combining an objective assessment of current progress with a clear discussion of existing limitations, this review outlines the key research directions that are likely to shape the future development of iron oxide nanoparticle-based therapies for glaucoma [14].

### 5.5. Metal–Organic Frameworks

Metal–organic frameworks (MOFs) have emerged as highly tunable porous materials for controlled drug delivery because their pore size, surface chemistry, and metal–ligand composition can be modified to regulate drug loading and release [55].

Sharabati et al. highlight the strong potential of metal–organic frameworks (MOFs) as advanced platforms for controlled drug delivery and biomedical applications, including neurodegenerative and ocular diseases. Although zinc is an essential trace element and serves as an obligate catalytic cofactor for endogenous copper/zinc superoxide dismutase (Cu/Zn-SOD1) [23,56], the assertion that zinc ions released from decomposing Zn-MOFs exert direct, therapeutic antioxidant protection in glaucomatous eyes remains hypothetical and currently unverified in in vivo ocular models [14,56]. Uncoordinated intracellular Zn^2+^ accumulation can paradoxically induce mitochondrial depolarization, impair electron transport, and accelerate retinal ganglion cell apoptosis [20,46]. Consequently, MOF formulations must be engineered using biologically benign metal nodes (such as Fe^3+^ or Ca^2+^) and thoroughly screened for metal-ion dissociation kinetics to prevent localized cytotoxicity [56,57]. Another important point highlighted in the review is that MOFs can be designed as stimuli-responsive systems, releasing their payload in response to specific biological conditions such as pH variations or the presence of certain biomarkers. In the case of glaucoma, this property could enable more precise and efficient drug release directly within the ocular environment, which differs significantly from systemic conditions. As noted above, however, any secondary contribution of released zinc to local antioxidant protection remains a proposed and currently unverified hypothesis rather than an established therapeutic mechanism and would require direct in vivo validation in glaucomatous models before it can be considered a rationale for MOF design [56].

**Figure 3:**
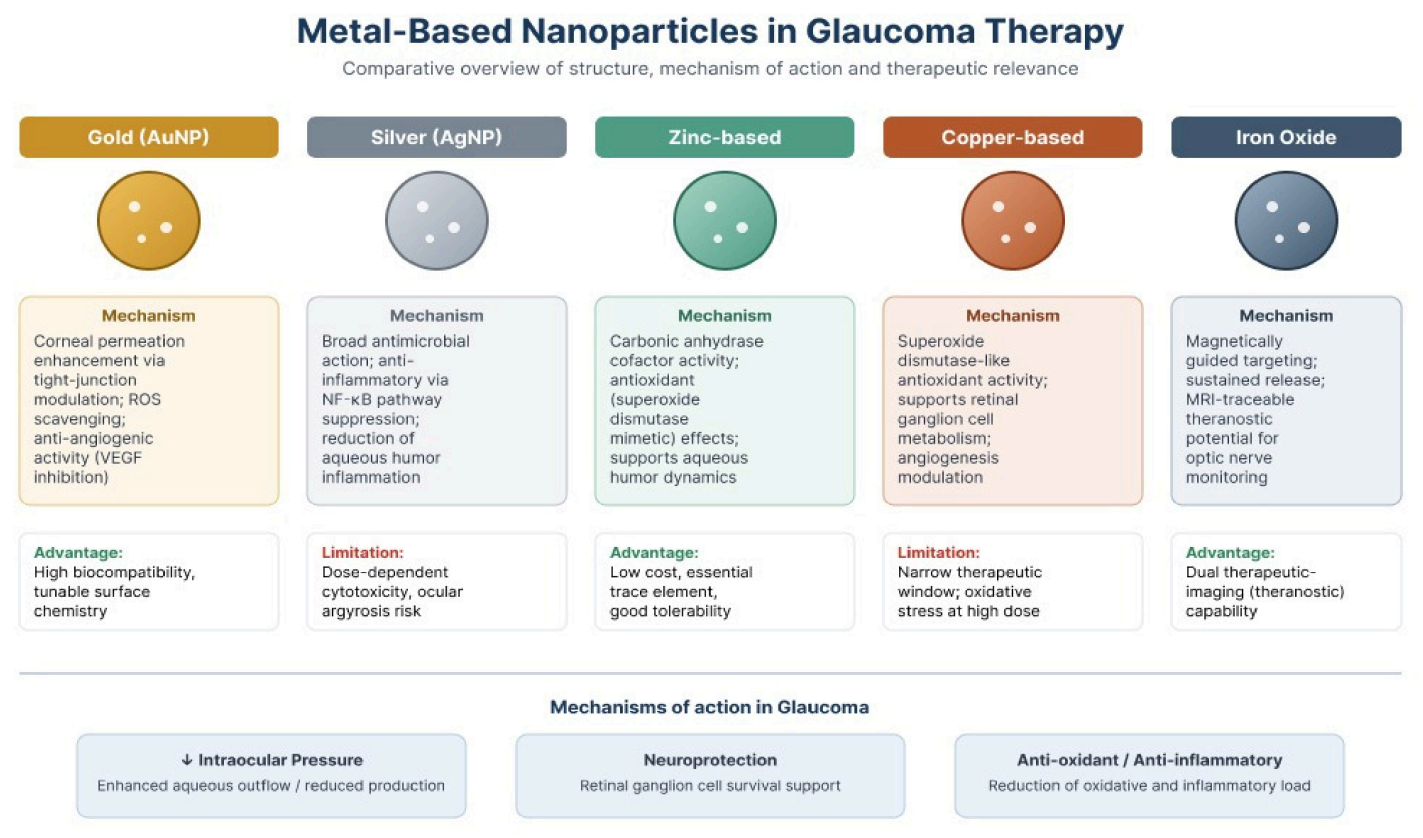
Schematic comparison of the five principal classes of metal-based nanoparticles. Color coding distinguishes nanoparticle classes and does not indicate comparative efficacy.

Kim et al. developed an NH_2_-MIL-88(Fe) formulation for topical delivery of brimonidine, which provided sustained drug release for up to 12 h, prolonged preocular retention for up to 4 h, and more than a two-fold increase in drug bioavailability and duration of action compared with marketed brimonidine eye drops [57]. Similarly, Gándara-Loe et al. evaluated several MOFs for brimonidine delivery and found high loading capacity for UiO-67 and MIL-100(Fe), with UiO-67 providing extended release for more than 12 days. These findings demonstrate the potential of MOFs to overcome rapid precorneal clearance and short drug exposure in glaucoma therapy [55]. (Figure 3).

Overall, metal-based nanoparticles offer several distinctive features for ocular drug delivery compared with conventional nanocarrier systems. Their physicochemical properties, surface chemistry, optical characteristics, and, in some cases, intrinsic biological activity may provide additional opportunities for controlled drug delivery and therapeutic effects. However, these advantages should be considered together with potential limitations related to ocular toxicity, metal-ion release, long-term accumulation, clearance, and reproducibility. In comparison, liposomes, polymeric nanoparticles, lipid-based systems, nanoemulsions, dendrimers, and hydrogels may offer advantages in terms of drug encapsulation, controlled release, biocompatibility, and formulation flexibility, although they may not provide the same intrinsic physicochemical or therapeutic properties associated with some metal-based platforms. Therefore, the selection of an appropriate nanocarrier for glaucoma therapy should depend on the therapeutic objective, drug characteristics, desired ocular residence time, safety profile, and level of available experimental evidence.

### 5.6. Nanotoxicology and the Risk of Iatrogenic Trabecular Occlusion

A critical yet frequently overlooked parameter in ophthalmic nanomedicine is the spatial dimension of the ocular drainage pathway [10,21]. In the human eye, aqueous humor drains primarily through the cribriform and juxtacanalicular regions of the trabecular meshwork, where functional intertrabecular pore diameters measure between 0.5 and 2.0 μm [1,27]. Chronic topical or intracameral administration of non-biodegradable inorganic nanomaterials (e.g., AuNPs, AgNPs, persistent silica, or robust MOFs) poses a substantial risk of physical pore occlusion [14,46]. In addition, repetitive uptake of non-degradable particles causes functional exhaustion of trabecular endothelial phagocytes [14,46]. Entrapped particles and aggregated cellular debris mechanically increase outflow resistance, paradoxically elevating intraocular pressure—the exact pathological hallmark these systems are engineered to prevent [1,46]. Furthermore, non-biodegradable particles that access the anterior chamber can settle onto the non-regenerative corneal endothelium, triggering membrane disruption and loss of corneal optical clarity [46]. Consequently, fully biodegradable systems (such as PLGA, polycaprolactone, and lipid nanoparticles) demonstrate superior clinical safety for chronic glaucoma management because their metabolic breakdown products (lactic acid, glycolic acid, endogenous fatty acids) are completely cleared without obstructing outflow pathways [16,34,58].

## 6. Nanocarrier-Based Ocular Drug Delivery Systems in Glaucoma

### 6.1. Liposomal Drug Delivery Systems in Glaucoma

Liposomes represent versatile lipid-based carriers for ocular drug delivery because their amphiphilic structure enables incorporation of both hydrophilic and lipophilic therapeutic compounds. The phospholipid bilayer can protect the encapsulated drug from premature degradation while also providing opportunities to modulate its release. In glaucoma therapy, these characteristics may improve drug retention at the ocular surface and facilitate exposure of target tissues compared with conventional topical formulations. However, factors such as physical instability, drug leakage, limited loading capacity for certain compounds, and challenges associated with large-scale manufacturing remain relevant considerations for their clinical translation [15].

Nordin et al. examined the potential of liposomes as topical drug delivery systems for glaucoma treatment. They noted that liposomal formulations can overcome many of the limitations associated with conventional eye drops by improving ocular bioavailability, increasing drug retention on the eye surface, and enhancing drug penetration through ocular barriers. In addition, liposomes provide controlled drug release, reduce the need for frequent administration, and may improve patient adherence to long-term therapy. The studies discussed in the review consistently showed that liposome-based formulations achieved a more sustained reduction in intraocular pressure while also offering potential neuroprotective effects on retinal ganglion cells. Overall, the authors suggest that topical liposomal drug delivery is a highly promising strategy for future glaucoma management, although further clinical validation and improvements in formulation and manufacturing are still needed [59].

Khallaf et al. developed a fasudil–phospholipid complex encapsulated in a thermosensitive in situ liposomal gel intended for topical administration in glaucoma. The authors demonstrated that this formulation significantly improved corneal permeability, mucoadhesion, and ocular surface retention time compared with conventional fasudil solution. Ex vivo and in vivo studies showed enhanced ocular bioavailability and a greater and more prolonged reduction in intraocular pressure in glaucomatous rabbits. Moreover, the formulation exhibited good ocular tolerability, with no signs of significant irritation, suggesting that the thermosensitive liposomal system represents a promising strategy for the efficient delivery of ROCK inhibitors in glaucoma treatment [31].

Liposomal delivery has also been investigated for prostaglandin analogs. Natarajan et al. developed phosphatidylcholine liposomes loaded with latanoprost and demonstrated sustained drug release together with prolonged IOP reduction following a single subconjunctival injection in rabbits. The IOP-lowering effect persisted for up to 90 days and exceeded that achieved with once-daily topical latanoprost, while no clinically evident ocular inflammation was observed. These findings illustrate the potential of liposomal systems to substantially extend the duration of action of prostaglandin analogs and reduce the need for repeated topical administration [60].

A multifunctional liposomal approach was evaluated by Fahmy et al., who co-encapsulated latanoprost with thymoquinone in an attempt to combine IOP lowering with additional tissue-protective effects. In glaucomatous rabbits, latanoprost-containing liposomes maintained significant IOP reduction for up to 84 h, while formulations containing thymoquinone improved some histopathological alterations. However, the formulations did not significantly reverse glaucoma-associated oxidative stress, highlighting that prolonged delivery does not necessarily translate into broader neuroprotective efficacy [61].

### 6.2. Polymeric Nanoparticles for Ocular Delivery

Polymeric nanoparticles are nanoscale drug-delivery systems composed of natural or synthetic polymers, designed to encapsulate, protect, and provide controlled release of therapeutic molecules. Due to their biodegradability, biocompatibility, and tunable surface properties, these systems can enhance drug transport across ocular barriers, improving bioavailability and therapeutic efficacy [16].

Polymeric nanoparticles provide several features that may be advantageous for ocular drug delivery, including protection of the active compound, modulation of drug release, and prolonged interaction with ocular tissues. Their nanoscale dimensions and tunable surface properties may also improve corneal permeation and ocular residence while allowing the use of biodegradable polymers such as poly(lactic-co-glycolic acid) (PLGA). In glaucoma, PLGA-based systems have been investigated for sustained delivery of carbonic anhydrase inhibitors. For example, phosphatidylserine-coated PLGA nanoparticles containing brinzolamide showed improved drug entrapment and corneal permeation and produced sustained IOP reduction after topical administration in experimental models. Other PLGA-based formulations have been designed to deliver multifunctional compounds with both pressure-lowering and cytoprotective activity, illustrating the possibility of extending polymeric nanocarriers beyond conventional IOP control [16].

Rawat et al. investigated nebivolol-loaded polycaprolactone nanoparticles incorporated into an in situ ocular gel to address the limited ocular exposure associated with conventional nebivolol formulations. The optimized system increased drug exposure in the aqueous humor and prolonged ocular residence compared with the conventional suspension. Pharmacokinetic and pharmacodynamic evaluations further indicated an approximately five-fold greater reduction in IOP with the nanoparticle formulation, together with sustained drug release. Together, these results point to the value of combining polymeric nanoparticles with in situ gels to extend therapeutic activity and reduce dosing frequency [62].

### 6.3. Solid Lipid Nanoparticles and Nanostructured Lipid Carriers

Solid Lipid Nanoparticles (SLNs) and Nanostructured Lipid Carriers (NLCs) are nanoscale colloidal drug-delivery systems developed using biocompatible and biodegradable lipids. SLNs consist of a solid lipid matrix capable of incorporating and protecting active pharmaceutical ingredients, whereas NLCs represent a second-generation lipid nanoparticle system composed of a mixture of solid and liquid lipids, allowing higher drug loading capacity, improved stability, and optimized drug-release properties [58].

NLCs may offer an additional formulation advantage over SLNs because the combination of solid and liquid lipids can generate a less ordered matrix with greater capacity for drug incorporation. These properties make both systems relevant candidates for prolonged ocular delivery; however, evidence supporting their use in glaucoma remains largely preclinical, and issues related to physical stability, drug loading, and reproducibility require further investigation.

Dang et al. investigated soft contact lenses incorporating PEGylated SLNs loaded with latanoprost as a means of prolonging ocular exposure. The nanoparticle-loaded lenses increased drug loading and provided sustained release for up to 96 h. In vivo experiments showed higher and more stable ocular drug concentrations than those obtained with conventional latanoprost eye drops, while histopathological evaluation indicated good ocular tolerability. These results support the use of SLN-loaded contact lenses as a potential long-acting alternative to repeated topical administration [32].

More recently, poly(2-ethyl-2-oxazoline)-coated SLNs were developed for timolol maleate delivery. The optimized nanoparticles showed high drug entrapment, sustained release, enhanced transcorneal and transscleral permeation, and a more prolonged IOP-lowering effect than the marketed formulation, while remaining non-irritant in ocular safety testing [34].

### 6.4. Nanoemulsions and Nanosuspensions

Nanoemulsions are colloidal systems composed of two immiscible liquid phases, where one phase is dispersed as nanometer-sized droplets stabilized by surfactants. Due to their small droplet size, these systems can improve the solubility, stability, and bioavailability of active compounds, enhancing the efficiency of ocular drug delivery. Nanosuspensions are colloidal systems consisting of nanometer-sized solid drug particles (generally 10–1000 nm) dispersed in an aqueous or other liquid medium and stabilized by agents that prevent particle aggregation. These systems are particularly designed for poorly water-soluble drugs, as reducing particle size increases surface area, enhances dissolution rate, and improves drug bioavailability. In ophthalmic therapy, nanosuspensions can increase drug concentration on the ocular surface, improve corneal penetration, and prolong therapeutic activity compared with conventional suspensions.

Nanoemulsions have attracted interest in ocular drug delivery because their small dispersed droplets provide a large interfacial area and can improve the solubilization and distribution of poorly water-soluble compounds. Their liquid nature may also facilitate formulation of hydrophobic therapeutic agents that are difficult to incorporate into conventional aqueous eye drops. In glaucoma therapy, nanoemulsions have been investigated as a means of increasing ocular residence and improving drug exposure while maintaining a suitable formulation for topical administration. Their potential benefits must nevertheless be considered alongside limitations such as physical instability, sensitivity to formulation composition, and the need to establish long-term ocular tolerability [45].

Pham et al. investigated a PLGA-based nanosuspension containing the hybrid molecule SA-10, designed to protect retinal ganglion cells against neuronal damage associated with glaucoma. The novelty of this study lies in combining a molecule with neuroprotective potential with a nano-based delivery system capable of improving its distribution and therapeutic efficiency at the ocular level. The authors observed that both free SA-10 and its PLGA nanosuspension formulation contributed to maintaining retinal ganglion cell viability and reducing experimentally induced neuronal injury. The use of the PLGA platform allowed improved dispersion of the active compound and enhanced retinal cell protection by limiting mechanisms associated with oxidative stress and inflammation. This is a meaningful distinction, since it suggests that nanoparticle-based therapies should not only focus on lowering intraocular pressure but also on directly protecting the neural structures affected by glaucoma. The authors therefore pointed to the potential of polymeric nanosuspensions as a modern strategy for developing neuroprotective therapies aimed at slowing disease progression [63].

### 6.5. Dendrimers and Advanced Nanocarriers

Dendrimers are three-dimensional polymeric nanostructures with a highly branched architecture and nanoscale size, consisting of a central core, repetitive units, and terminal functional groups. Due to their well-defined structure, large surface area, and multiple functional groups, dendrimers can encapsulate or bind therapeutic molecules, improving their solubility, stability, and delivery to target tissues. These properties make them promising platforms for controlled drug delivery [64].

The formulation described by Wang et al. combined dendrimer-based particles with a gel matrix to improve topical delivery of antiglaucoma agents. The highly branched dendrimer structure and extensive surface functionality provided opportunities for drug incorporation, while the gel component supported prolonged retention and controlled release. Experimental results indicated improved ocular-surface retention, corneal penetration, and a more sustained reduction in IOP compared with conventional formulations. The results indicate that dendrimer–gel systems may be useful for reducing the frequency of topical administration [65].

Pitha et al. developed an innovative dendrimer–drug conjugate system designed to reduce neuroinflammation and protect retinal ganglion cells in glaucoma. Unlike conventional therapies that mainly focus on lowering intraocular pressure, this strategy targets one of the key mechanisms involved in disease progression, namely excessive activation of retinal microglia. Microglia are the resident immune cells of the central nervous system and, under pathological conditions such as glaucoma, they can adopt a pro-inflammatory phenotype, releasing cytokines and oxidative mediators that contribute to retinal ganglion cell damage and progressive vision loss [35].

To address this mechanism, the authors used PAMAM (polyamidoamine) dendrimers as delivery platforms for a therapeutic agent with anti-inflammatory activity. Due to their three-dimensional, highly branched structure and the presence of multiple functional groups on their surface, dendrimers allow efficient drug conjugation and can promote the accumulation of therapeutic molecules in inflamed tissues. Therefore, the dendrimer–drug conjugate was designed to selectively target activated microglial cells, improving therapeutic efficiency while reducing non-specific exposure of surrounding tissues. The obtained results demonstrated that this nanotechnology-based platform reduced microglial activation and decreased the production of inflammatory mediators, thereby limiting neurotoxic processes associated with glaucoma. Taken together, this work shows that dendrimers can serve not only as drug carriers but also as advanced tools for developing targeted therapies directed toward cellular mechanisms involved in neurodegeneration [35].

Cho et al. developed a dendrimer-based formulation of triamcinolone acetonide (D-TA) designed to improve the delivery of corticosteroid therapy to retinal tissues affected by ischemic injury. The main innovation of this approach was the conjugation of triamcinolone acetonide with hydroxyl-terminated polyamidoamine (PAMAM) dendrimers, creating a targeted nanocarrier capable of enhancing drug accumulation in inflamed retinal areas while reducing the limitations associated with conventional corticosteroid administration. The study revealed that the dendrimer–triamcinolone acetonide system significantly reduced neuroinflammatory responses by limiting microglial activation and decreasing the expression of inflammatory mediators. In addition, the formulation reduced pathological angiogenesis and improved neuroretinal function by protecting retinal neurons from ischemic damage. These effects were associated with the ability of dendrimers to preferentially accumulate in injured tissues and deliver the therapeutic agent directly to sites of inflammation.

Although this study focused on ischemic retinopathy rather than glaucoma, its relevance to glaucoma therapy lies in the shared involvement of chronic neuroinflammation, oxidative stress, and retinal neuronal loss. The results support the potential use of dendrimer-based corticosteroid delivery systems as future neuroprotective strategies aimed at preserving retinal function in ocular neurodegenerative diseases [66].

Beyond conventional nanoparticle preparation, advanced formulation technologies such as electrospinning and cyclodextrin-based molecular assemblies illustrate how carrier architecture can be engineered to improve drug incorporation, stability, and release behavior [67].

## 7. Cyclodextrin-Based Ocular Drug Delivery Systems

Cyclodextrins are cyclic oligosaccharides characterized by a hydrophilic outer surface and a relatively hydrophobic internal cavity, which enables the formation of inclusion complexes with a wide range of poorly water-soluble drugs. Through these interactions, cyclodextrin-based systems can improve drug solubility, stability, incorporation into delivery matrices, and release behavior [67].

Cyclodextrins are particularly relevant to glaucoma because their hydrophilic exterior and relatively lipophilic cavity enable the formation of inclusion complexes with poorly water-soluble antiglaucoma drugs. A particularly relevant example is dorzolamide, for which γ-cyclodextrin-based microparticle and nanoparticle eye drops have been developed to prolong ocular residence and sustain drug availability. Clinical evaluation of γ-cyclodextrin nanoparticle eye drops demonstrated their feasibility for IOP control, illustrating the potential of cyclodextrin self-assembly as an alternative strategy for delivering carbonic anhydrase inhibitors in glaucoma [68].

Cyclodextrins have also been explored for latanoprost, a lipophilic prostaglandin analog whose formulation is complicated by poor aqueous solubility and limited chemical stability. Complexation with selected β-cyclodextrin derivatives improved latanoprost solubilization and protected its ester functionality during storage, demonstrating how cyclodextrins can contribute not only to drug solubility but also to formulation stability [69].

Importantly, cyclodextrin complexation does not invariably improve ocular bioavailability; excessive drug binding or unsuitable formulation conditions may reduce the fraction of free drug available for corneal permeation.

## 8. Hydrogel-Based Nanoformulation for Ocular Delivery

Hydrogel is a three-dimensional hydrophilic polymer network capable of absorbing and retaining large amounts of water or biological fluids while maintaining its structural integrity. Due to their high biocompatibility, mucoadhesive properties, and ability to provide controlled and sustained drug release, hydrogels are widely used as drug delivery systems. In ocular applications, they prolong the residence time of drugs on the eye surface, enhance ocular bioavailability, reduce precorneal drug loss, and improve therapeutic efficacy while decreasing the frequency of administration [70].

### Nanoparticle-Loaded Hydrogels

Kumar et al. developed Eudragit S100 nanoparticle-loaded hydrogel contact lenses for the sustained ocular delivery of levobunolol, aiming to overcome the poor bioavailability and frequent dosing associated with conventional glaucoma eye drops. The optimized nanoparticles exhibited high drug encapsulation efficiency, suitable nanoscale dimensions, and were successfully incorporated into soft contact lenses without compromising their transparency, oxygen permeability, or physical properties. The therapeutic lenses provided a controlled drug release for up to 12 days, significantly longer than drug solution-loaded lenses, while ex vivo studies demonstrated enhanced transcorneal permeation compared with commercially available eye drops [71].

**Figure 4:**
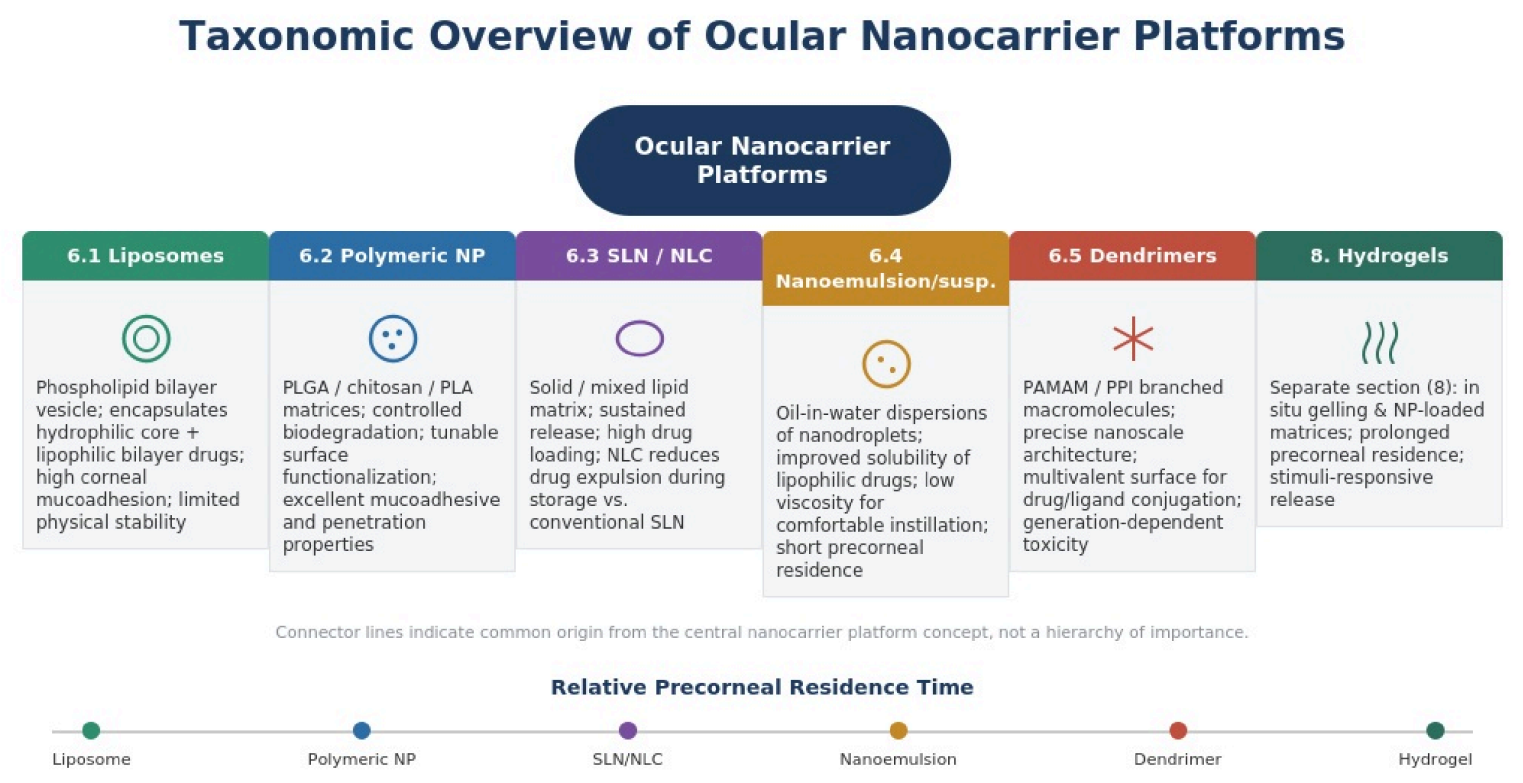
Taxonomic overview of the principal nanocarrier platforms (liposomes, polymeric nanoparticles, SLN/NLC, nanoemulsions/nanosuspensions, dendrimers, and nanoparticle-loaded hydrogels) and their relative precorneal residence time. Color coding distinguishes nanocarrier classes and does not indicate comparative efficacy; “8. Hydrogels” corresponds to Section 8 of the manuscript.

Åhlén et al. investigated the potential of nanoparticle-loaded hydrogels as enzyme-responsive ocular drug delivery systems, focusing on the development of a platform capable of releasing therapeutic agents in response to biological triggers present in ocular tissues. The authors explored how incorporating nanoparticles into hydrogel matrices can improve drug stability, prolong residence time on the ocular surface, and enable more controlled and localized drug release compared with conventional ophthalmic formulations [72]. (Figure 4).

Given the large number and heterogeneity of ocular nanoformulations reported in the literature, a selection of representative systems was chosen to illustrate the main formulation strategies discussed in this review. Table 2 summarizes these representative nanoformulations in terms of drug loading or encapsulation efficiency, particle size, administration route and experimental model, and key release or therapeutic outcomes.

## 9. Mechanism of Action

A common limitation in ocular nanomedicine reviews is the conflation of anterior segment hypotensive therapies with posterior segment neuroprotective interventions [14,73]. Effective carrier formulation requires design features matched to the targeted anatomical compartment [10,11]:

1. Anterior Segment Targeting (Hypotensive Therapy): target structures comprise the trabecular meshwork, Schlemm’s canal, and the ciliary body non-pigmented epithelium [1,27]. Delivery requires transcorneal permeation following topical administration [10]. Formulations must prioritize mucoadhesive properties (e.g., chitosan or in situ gelling polymers) and positive or amphiphilic surface charges to extend precorneal retention, resist nasolacrimal flushing, and sustain the release of IOP-lowering agents (such as prostaglandin analogs, ROCK inhibitors, and carbonic anhydrase inhibitors) [12,31,32].
2. Posterior Segment Targeting (Neuroprotection): Target cells comprise retinal ganglion cells, their unmyelinated axons at the optic nerve head, and reactive retinal microglia and macroglia [3,8,9]. Topical delivery to these posterior structures is largely ineffective, as retro-directional aqueous humor outflow, rapid choroidal blood clearance, and the blood-retinal barrier restrict posterior drug penetration to less than 0.001% of the instilled topical dose [10,11]. Posterior neuroprotection therefore relies on subconjunctival/transscleral depots, suprachoroidal injections, or intravitreal delivery [11,73]. Intravitreally injected carriers must possess a slightly negative or densely PEGylated neutral surface charge to prevent electrostatic immobilization within the polyanionic hyaluronic acid meshwork of the vitreous, allowing sustained diffusion of neuroprotective agents (e.g., BDNF, coenzyme Q10, siRNA, or dendrimer-antioxidant conjugates) directly to the inner limiting membrane and RGC layer [24,35,66,73].

### 9.1. Intraocular Pressure Reduction

Elevated intraocular pressure (IOP) is the primary modifiable risk factor for glaucoma progression and remains the main therapeutic target in glaucoma management. Under physiological conditions, IOP is regulated by a balance between aqueous humor production by the ciliary body and its drainage through the trabecular meshwork and uveoscleral pathways. Increased resistance to aqueous humor outflow disrupts this balance, leading to elevated IOP, optic nerve damage, and progressive loss of retinal ganglion cells.

Current pharmacological therapies reduce IOP by either decreasing aqueous humor production or enhancing its outflow. β-Blockers and carbonic anhydrase inhibitors reduce aqueous humor secretion, whereas prostaglandin analogs increase uveoscleral outflow. Cholinergic agonists and Rho kinase (ROCK) inhibitors improve drainage through the trabecular meshwork, while α-2 adrenergic agonists exert both mechanisms by reducing aqueous humor production and promoting uveoscleral outflow. Recent advances focus on sustained drug-delivery systems, including hydrogels, nanoparticles, implants, and drug-eluting contact lenses, which provide prolonged drug release, improve ocular bioavailability, and maintain stable IOP while reducing dosing frequency and improving patient adherence [74].

### 9.2. Neuroprotection

Beyond lowering intraocular pressure, nanocarrier-based systems may facilitate therapies aimed at protecting retinal ganglion cells from glaucomatous damage. Their ability to improve drug stability, prolong ocular residence, and modify tissue distribution may increase exposure of neuroprotective compounds to relevant ocular targets. Experimental studies involving antioxidant, anti-inflammatory, and other neuroprotective agents have reported beneficial effects that cannot necessarily be attributed solely to IOP reduction. This lends support to further investigation of nanotechnology-based approaches as complementary strategies for limiting neuronal degeneration in glaucoma.

Zinc-chelator-loaded PLGA nanoparticles provide an example of a nanocarrier investigated for neuroprotective activity. In optic nerve crush and acute ocular hypertension models, the formulation was associated with neuroprotective and axon-regenerative effects that persisted independently of IOP reduction, supporting the possibility of a direct protective mechanism [73].

### 9.3. Anti-Inflammatory and Antioxidant Effects

Oxidative stress and inflammatory signaling are implicated in several processes associated with glaucoma progression and may contribute to cellular dysfunction in both the anterior and posterior segments of the eye. In the trabecular meshwork, oxidative damage can impair cellular homeostasis and interfere with aqueous humor outflow. Nanoformulations may contribute to these therapeutic strategies by improving the stability, solubility, and ocular delivery of antioxidant or anti-inflammatory compounds. One particularly telling example: a single intravitreal dose of a dendrimer-conjugated N-acetylcysteine, given a week after pressure elevation, markedly reduced the transcription of pro-inflammatory markers such as IL-1β, IL-6, and MCP-1, while measurably improving retinal ganglion cell survival compared with the free drug [24].

**Figure 5:**
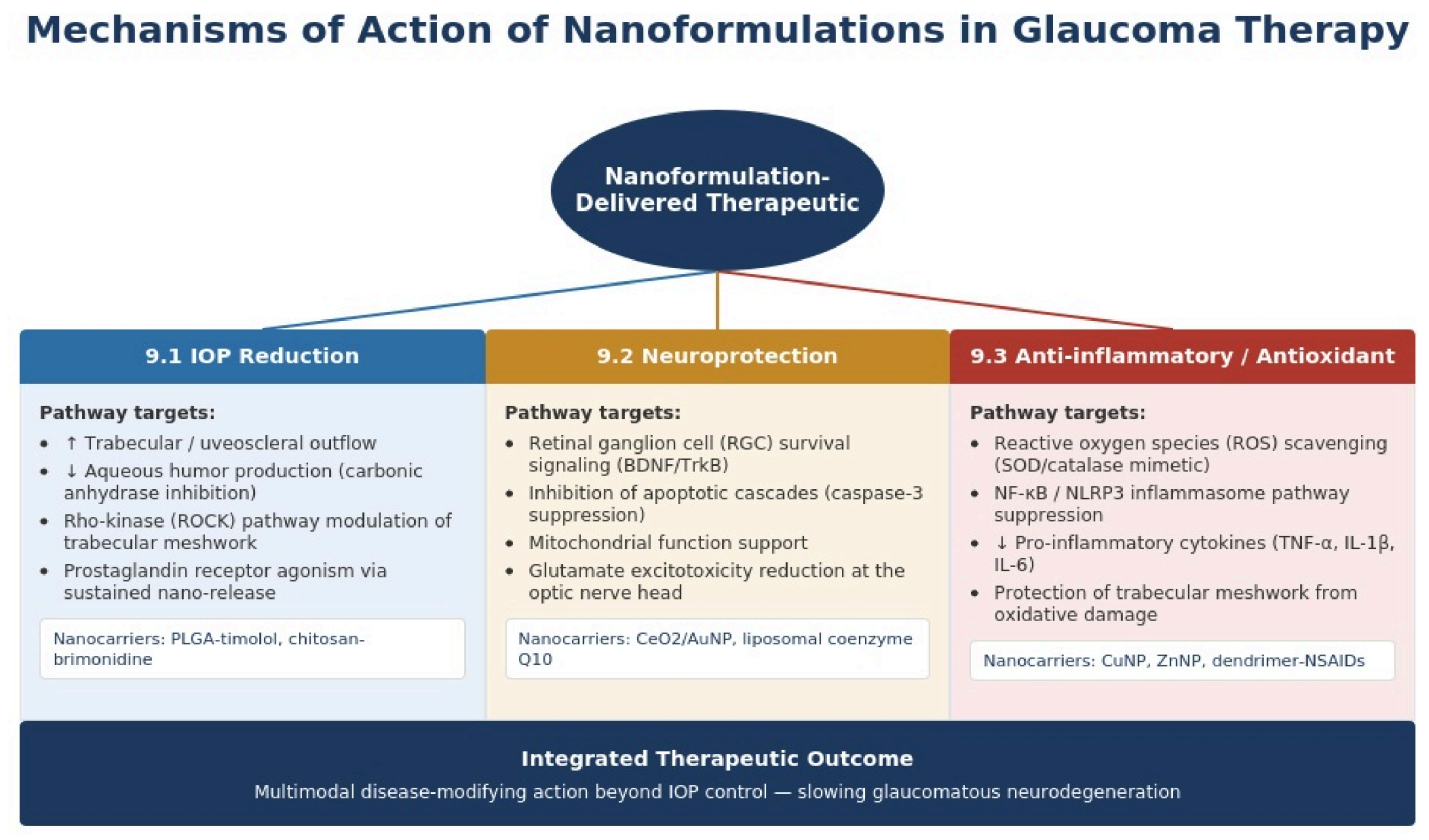
Molecular and cellular mechanisms of action of nanoformulation-based glaucoma therapy.

As illustrated in Figure 5, no single nanoformulation platform provides an optimal solution for all requirements of glaucoma therapy. Lipid- and polymer-based systems may be particularly advantageous for controlled drug release and formulation flexibility, whereas hydrogels can substantially improve ocular residence. Metal-based nanoparticles offer additional physicochemical and potentially multifunctional properties, but their long-term safety and biological fate require particular attention. The optimal platform should therefore be selected according to the therapeutic objective and the physicochemical and biological characteristics of the drug.

## 10. Advantages and Limitations of Nanoformulations

The principal advantage of nanoformulations lies in their ability to modify the spatial and temporal distribution of ophthalmic drugs, potentially increasing ocular exposure while reducing premature elimination.

Zhou et al. reported that nanocarriers improve ocular bioavailability through prolonged precorneal residence time, enhanced corneal penetration, and protection of encapsulated drugs from degradation, allowing a greater proportion of the administered dose to reach target tissues. Their ability to provide sustained and controlled drug release maintains therapeutic drug concentrations for extended periods, reducing dosing frequency and improving patient adherence to long-term treatment. Moreover, nanoformulations can minimize systemic drug absorption and local ocular irritation by increasing drug retention at the site of action, thereby improving the overall safety profile of antiglaucoma medications. The authors also noted the potential of surface-functionalized nanocarriers for targeted ocular delivery, as well as their versatility in enabling combination therapy and the development of multifunctional systems with diagnostic and therapeutic capabilities. Collectively, these advantages position nanoformulations as a promising platform for improving the efficacy, safety, and precision of glaucoma treatment [36].

Kundu et al. noted that these systems provide improved spatial and temporal control over drug administration, allowing therapeutic molecules to be released only when required. This mechanism reduces premature drug release, improves treatment precision, and minimizes unnecessary exposure of ocular tissues to active compounds. Nanocarriers provide a protective environment for encapsulated drugs, preventing degradation caused by enzymatic activity, oxidation, or unfavorable physicochemical conditions. Encapsulation has similarly been reported to improve drug stability and maintain therapeutic activity during administration, an advantage that is particularly relevant for unstable molecules or poorly soluble compounds requiring improved formulation strategies to achieve adequate ocular delivery. Nanoformulations offer the possibility of incorporating multiple therapeutic agents within a single carrier, providing an opportunity for combination therapy in glaucoma management. Multifunctional nanocarriers can simultaneously deliver drugs with different mechanisms of action, potentially improving therapeutic outcomes while simplifying treatment protocols. Some nanoplatforms also allow integration of diagnostic functions, creating theranostic systems capable of monitoring drug distribution [53].

Although nanoformulations have demonstrated promising results in preclinical studies, one of the main limitations remains the lack of extensive clinical validation. Zhai et al. highlighted that many nanomedicine-based glaucoma therapies remain at the experimental stage, with limited evidence regarding long-term efficacy, safety, and therapeutic outcomes in humans. Differences between animal models and human ocular physiology represent an important challenge, as nanoparticle distribution, clearance, and biological interactions may vary significantly. Therefore, further clinical trials are required to confirm whether the benefits observed in laboratory studies can be successfully translated into routine glaucoma management. A major limitation associated with nanoformulations is the complexity of their production and difficulties in achieving large-scale manufacturing. Kundu et al. demonstrated that maintaining consistent nanoparticle size, surface characteristics, drug-loading capacity, and release behavior during industrial production remains challenging. Small variations in formulation parameters may significantly influence therapeutic performance and safety [14,53].

Despite their therapeutic advantages, nanoparticles may raise concerns regarding ocular toxicity and long-term biological interactions. According to recent reviews, the accumulation of nanomaterials within ocular tissues, particularly after repeated administration, requires careful evaluation. Factors such as particle composition, size, surface charge, and degradation products may influence inflammatory responses, oxidative stress, or cellular toxicity. Therefore, the development of biodegradable and biocompatible nanocarriers remains essential for ensuring safe long-term glaucoma treatment.

Compared with traditional eye drops, nanoformulations often require advanced materials, specialized equipment, and complex preparation methods, leading to increased production costs. This economic limitation may restrict their widespread application, particularly in healthcare systems where cost-effectiveness is a major consideration. Although nanotechnology can improve therapeutic outcomes, reducing manufacturing expenses remains necessary to facilitate large-scale clinical implementation [75].

### Translational Bottlenecks in Ocular Nanomedicine

Despite compelling preclinical results, the translation of ocular nanoformulations from benchtop research to clinical practice remains constrained by critical pharmaceutical engineering, quality-control, and regulatory hurdles [14,53,75].

First, scalable Good Manufacturing Practice (GMP) production remains a major bottleneck; formulation techniques commonly used in laboratory settings—such as thin-film hydration, probe sonication, and solvent displacement—are poorly suited for industrial batch sizes exceeding 100 L [14,53]. Large-scale synthesis frequently compromises batch-to-batch reproducibility, resulting in broader polydispersity indices (PDI > 0.3), unpredicted particle agglomeration, and inconsistent drug loading or release profiles [53,75]. Continuous microfluidic fabrication is currently being explored to provide precise hydrodynamic flow focusing and narrow size distributions during industrial scale-up [53].

Second, terminal sterilization poses formidable challenges, as all ophthalmic preparations must ensure absolute sterility without compromising carrier stability [10,40]. Standard autoclaving exceeds the phase transition temperatures of phospholipids and solid lipids, causing liposomal membrane rupture, particle coalescence, and thermal hydrolysis of biodegradable polymers such as PLGA [15,58]. Gamma-irradiation generates radiolytic free radicals that chemically degrade encapsulated active moieties and alter polymeric chains, while conventional 0.22 μm sterile membrane filtration is viable only for rigid nanocarriers below 150 nm, as larger particles, nanosuspensions, and in situ gelling matrices rapidly clog filter pores [45,53,75]. Third, aqueous colloidal systems are susceptible to physical destabilization during storage via Ostwald ripening, drug expulsion, and sedimentation [45,58]. Lyophilization with cryoprotectants (e.g., trehalose, mannitol, or sucrose at 5–10% *w*/*v*) is often mandatory to guarantee acceptable commercial shelf-life (>24 months) without compromising reconstitution redispersibility [14,53].

Finally, regarding regulatory pathways and human clinical trials, translation remains predominantly restricted to non-metallic biodegradable platforms [14,15]. Notable clinical milestones include the FDA-approved Durysta^®^ (an intracameral biodegradable PLGA rod sustaining bimatoprost release over 4–6 months), commercial cationic nanoemulsions such as Cationorm^®^, and preservative-free cationic latanoprost emulsions evaluated in completed Phase II (Catiolanze) and Phase III multicenter clinical trials [42,43]. In contrast, inorganic and metallic nanoparticles remain entirely within the preclinical exploratory phase, as unresolved questions regarding ocular nanotoxicology, chronic clearance pathways, and heavy-metal tissue accumulation continue to impede their entry into active Phase I–III clinical trials [14,46,75].

## 11. Future Perspectives

Future research in glaucoma nanomedicine is likely to focus on improving the specificity, duration, and safety of ocular drug delivery. Stimuli-responsive nanocarriers could provide drug release in response to physicochemical or disease-associated signals, while ligand-functionalized systems may improve interaction with selected cellular or tissue targets. Nanotechnology may also facilitate the delivery of nucleic acids and other biological therapeutics that are difficult to administer using conventional formulations. Progress toward clinical translation will depend on reproducible manufacturing, long-term ocular safety, predictable pharmacokinetics, and demonstration of therapeutic benefit in clinically relevant models [76].

**Figure 6:**
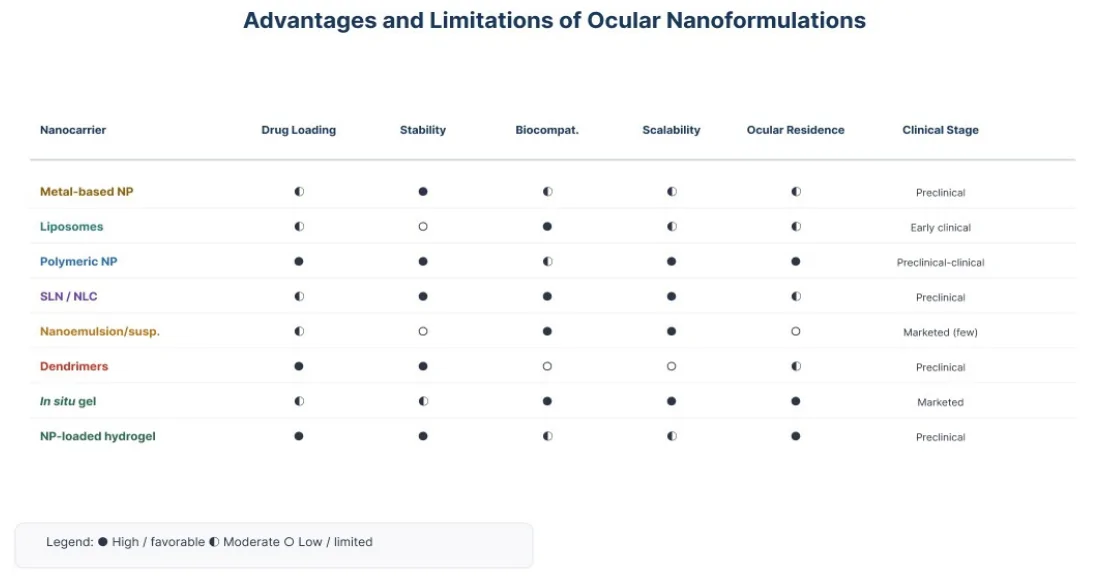
Qualitative comparative matrix summarizing the advantages and limitations of the principal nanoformulation classes discussed in this review. The matrix is complete, and the legend defines the qualitative symbols used to compare the listed characteristics.

Recent advances in non-viral gene delivery have drawn attention to the potential of CRISPR/Cas9-based therapies for future ocular disease management. Hua et al. reviewed the use of lipid nanoparticles and other non-viral nanocarriers for safe and efficient delivery of CRISPR components, emphasizing their high biocompatibility, transient gene expression, and reduced risk of off-target effects compared with viral vectors. The authors also stressed the importance of tailoring nanoparticle design to overcome ocular anatomical barriers, suggesting that these technologies may enable long-term, targeted treatment strategies for glaucoma by improving gene delivery to tissues involved in aqueous humor outflow [77]. (Figure 6).

## 12. Conclusions

Nanoformulations provide several opportunities to address important limitations associated with conventional glaucoma therapy, particularly short precorneal residence time, limited ocular bioavailability, and frequent dosing requirements. A broad range of platforms, including liposomes, polymeric nanoparticles, solid lipid nanoparticles, nanostructured lipid carriers, nanoemulsions, nanosuspensions, dendrimers, and hydrogels, have demonstrated potential to improve drug retention, controlled release, ocular exposure, and therapeutic efficacy. Within this broader landscape, metal-based nanoparticles represent a distinctive group of nanoplatforms because their physicochemical and, in some cases, intrinsic biological properties may provide additional opportunities for multifunctional ocular therapy. However, these potential advantages must be considered alongside specific challenges related to toxicity, long-term accumulation, clearance, reproducibility, and safety. Therefore, metal-based nanoparticles should not be considered universally superior to other nanocarriers; rather, their suitability depends on the therapeutic objective, drug characteristics, formulation properties, and intended route of administration. Despite promising results reported across these nanoformulation classes, current evidence remains heterogeneous and is predominantly based on in vitro and preclinical studies. Future research should prioritize standardized physicochemical characterization, long-term ocular safety assessment, pharmacokinetic and biodistribution studies, reproducible manufacturing, and direct comparison between different nanocarrier platforms and established glaucoma treatments. Ultimately, the clinical value of glaucoma nanomedicine will depend on whether the advantages observed in experimental models translate into sustained therapeutic efficacy, improved treatment adherence, and meaningful preservation of visual function.

## 13. Limitations

Interpreting the available literature requires caution because the evidence supporting ocular nanoformulations for glaucoma is highly heterogeneous. Many studies rely on small experimental cohorts, short observation periods, and acute or normotensive animal models that do not fully reproduce the chronic and progressive nature of human glaucoma. Moreover, methodological features that reduce experimental bias, including masked intraocular pressure assessment, allocation concealment, predefined exclusion criteria, and independent replication, are inconsistently reported. Consequently, efficacy signals should be interpreted as proof-of-concept rather than as evidence of established translational superiority.

## Figures and Tables

**Table 1:** Conventional ocular drug delivery systems: advantages, limitations, and clinical impact.

System	Advantages	Limitations	Clinical Impact	Representative Commercial Products
Eye drops	Simple, most widely used ophthalmic formulation	Rapid tear clearance; <5% bioavailability; frequent dosing; preservative toxicity	Poor adherence in chronic therapy	Timoptic^®^ (timolol); Xalatan^®^ (latanoprost); Alphagan P^®^ (brimonidine)
Emulsions	Better solubility for hydrophobic drugs; sustained release	Formulation instability; uneven distribution; surfactant-induced irritation	Unpredictable therapeutic response	Restasis^®^ (cyclosporine 0.05% ophthalmic emulsion)—emulsion technology example (dry eye; not IOP-lowering)
Suspensions	Suitable for hydrophobic drugs; prolonged retention	Sedimentation; inconsistent dosing; particle-induced irritation	Variable efficacy; reduced compliance	Azopt^®^ (brinzolamide 1% suspension); Betoptic S^®^ (betaxolol 0.25% suspension)
Solutions	Sterile, simple, fast onset of action	Rapid clearance; low absorption; preservative toxicity	Requires frequent re-administration	Xalatan^®^ (latanoprost); Cosopt^®^ (dorzolamide/timolol); Betoptic^®^ (betaxolol)
Ointments	Prolonged ocular contact; sustained release	Blurred vision; greasy sensation; imprecise dosing	Low patient acceptance	Pilopine HS^®^ (pilocarpine 4% gel, bedtime use)

**Table 2:** Comparative characteristics and experimental evidence for representative nanoformulations investigated in glaucoma drug delivery.

Nanocarrier/Platform	Drug	EE (%)	Drug Loading/Loading Capacity	Particle Size	Administration/Model	Key Experimental Finding	Ref.
Liposomal thermosensitive in situ gel	Fasudil	77.9 ± 0.5 (gel); 78.6 ± 0.3 (liposomal dispersion)	26.9 ± 0.25	132.5 ± 1.6 nm (liposomal dispersion)	Topical; glaucomatous rabbits	Sustained release; improved ocular bioavailability and greater/prolonged IOP reduction versus drug solution	[31]
PLGA-coated gold nanospheres/nanorods	Carbonic anhydrase inhibitor	>98	Not reported	569.3 ± 25.6 nm/726.9 ± 57.7 nm	Topical; rabbit glaucoma model	Greater IOP reduction than marketed eye drops; spherical particles showed better ocular retention than nanorods	[48]
NH_2_-MIL-88(Fe) MOF	Brimonidine	Not expressed as EE%	121.3 µg/mg carrier (~12.13% *w*/*w*)	0.5–3 µm	Topical; healthy rabbits	Sustained release up to 12 h; preocular retention up to 4 h; >2-fold increase in bioavailability/activity period	[57]
UiO-67/MIL-100(Fe) MOFs	Brimonidine tartrate	Not expressed as EE%	Up to 50–60 wt%	Submicron particles	In vitro ocular-delivery evaluation; retinal-cell cytotoxicity testing	UiO-67 showed extended release >12 days	[55]
EggPC liposomes	Latanoprost	94 ± 5	Drug/lipid molar ratio = 0.181; final drug concentration >1 mg/mL	109 ± 18 nm	Single subconjunctival injection; rabbit eyes	60% released by 14 days in vitro; sustained IOP lowering >90 days	[60]
DPPC liposomes	Latanoprost/thymoquinone	LAT 88; TQ 92	DL: LAT 18%; TQ 20%; LAT+TQ co-loaded liposomes 25%	<10 µm	Subconjunctival injection in glaucoma-induced rabbits	LAT and LAT+TQ liposomes reduced IOP significantly up to 84 h	[61]
Poly(2-ethyl-2-oxazoline)-coated SLNs	Timolol maleate	96.2 ± 0.85	Not reported	107 ± 17 nm	Ocular administration in rabbits; ex vivo evaluation on excised goat cornea and sclera	Sustained release over ~12 h; enhanced corneal/scleral permeation and prolonged IOP reduction	[34]
PCL polymeric nanoparticles loaded into in situ gel	Nebivolol	96.7 ± 0.3	28.8 ± 2.4%	270.9 ± 6.3 nm	Ocular; rabbits	In situ gel increased aqueous-humor exposure and prolonged ocular residence over 24 h; sustained IOP reduction	[62]
Eudragit S100 nanoparticles incorporated into contact lenses	Levobunolol	86.99 ± 1.9	Contact-lens drug uptake: 0.5676 mg after 24 h soaking	102.61 ± 3.92 nm	Ex vivo goat cornea	84.33 ± 0.34% released over 12 days versus ~3 days for drug-solution-loaded lenses	[71]

EE, encapsulation/entrapment efficiency; DL, drug loading; IOP, intraocular pressure; MOF, metal–organic framework; SLN, solid lipid nanoparticle; PCL, polycaprolactone; PLGA, poly(lactic-co-glycolic acid); DPPC,1,2-dipalmitoyl-sn-glycero-3-phosphocholine.

## Data Availability

No new data were created or analyzed in this study. Data sharing is not applicable to this article.

## Abbreviations

The following abbreviations are used in this manuscript:

IOP	Intraocular pressure
ROS	Reactive oxygen species
TNF-α	Tumor necrosis factor-alpha
IL-1β	Interleukin-1 beta
IL-6	Interleukin-6
NMDA	N-methyl-D-aspartate
ROCK	Rho kinase
AuNP	Gold nanoparticle
AgNP	Silver nanoparticle
CuNP	Copper-based nanoparticle
MOF	Metal–organic framework
PLGA	Poly(lactic-co-glycolic acid)
SLN	Solid lipid nanoparticle
NLC	Nanostructured lipid carrier
PAMAM	Polyamidoamine (dendrimer)
D-TA	Dendrimer-triamcinolone acetonide conjugate
